# Radiosurgery for ventricular tachycardia (RAVENTA): interim analysis of a multicenter multiplatform feasibility trial

**DOI:** 10.1007/s00066-023-02091-9

**Published:** 2023-06-07

**Authors:** David Krug, Adrian Zaman, Lina Eidinger, Melanie Grehn, Judit Boda-Heggemann, Boris Rudic, Felix Mehrhof, Leif-Hendrik Boldt, Stephan Hohmann, Roland Merten, Daniel Buergy, Jens Fleckenstein, Anne Kluge, Annette Rogge, Marcus Both, Dirk Rades, Roland Richard Tilz, Denise Olbrich, Inke R. König, Frank-Andre Siebert, Achim Schweikard, Reinhard Vonthein, Hendrik Bonnemeier, Jürgen Dunst, Oliver Blanck

**Affiliations:** 1 Klinik für Strahlentherapie, Universitätsklinikum Schleswig-Holstein, Campus Kiel, Arnold-Heller-Straße 3, Haus L, 24105 Kiel, Germany; 2 Klinik für Innere Medizin III, Kardiologie, Abteilung für Elektrophysiologie und Rhythmologie, Universitätsklinikum Schleswig-Holstein, Kiel, Germany; 3 Universitätsmedizin Mannheim, Klinik für Strahlentherapie und Radioonkologie, Medizinische Fakultät Mannheim, Universität Heidelberg, Mannheim, Germany; 4 Universitätsmedizin Mannheim, Medizinische Klinik I, Abteilung für Elektrophysiologie und Rhythmologie, Medizinische Fakultät Mannheim, Universität Heidelberg, Mannheim, Germany; 5 Klinik für Radioonkologie und Strahlentherapie, Charité Universitätsmedizin Berlin, Berlin, Germany; 6 Medizinische Klinik mit Schwerpunkt Kardiologie (CVK), Abteilung für Elektrophysiologie und Rhythmologie, Charité Universitätsmedizin Berlin, Berlin, Germany; 7 DZHK (German Centre for Cardiovascular Research), partner site Berlin, Berlin, Germany; 8 Hannover Herzrhythmus Centrum, Klinik für Kardiologie und Angiologie, Medizinische Hochschule Hannover, Hannover, Germany; 9 Klinik für Strahlentherapie und Spezielle Onkologie, Medizinische Hochschule Hannover, Hannover, Germany; 10 Klinisches Ethikkomitee, Universitätsklinikum Schleswig-Holstein, Kiel, Germany; 11 Klinik für Radiologie und Neuroradiologie, Universitätsklinikum Schleswig-Holstein, Kiel, Germany; 12 Klinik für Strahlentherapie, Universitätsklinikum Schleswig-Holstein, Lübeck, Germany; 13 Klinik für Rhythmologie, Universitätsklinikum Schleswig-Holstein, Lübeck, Germany; 14 Zentrum für Klinische Studien, Universität zu Lübeck, Lübeck, Germany; 15 Institut für Medizinische Biometrie und Statistik, Universitätsklinikum Schleswig-Holstein, Lübeck, Germany; 16 Institut für Robotik und Kognitive Systeme, Universität zu Lübeck, Lübeck, Germany; 17 Klinik für Kardiologie, Helios Klinik Cuxhaven, Cuxhaven, Germany

**Keywords:** Stereotactic arrythmia radioablation, Stereotactic body radiotherapy, Cardiac arrhythmia, Implantable cardioverter defibrillator, Structural heart disease

## Abstract

**Background:**

Single-session cardiac stereotactic radiation therapy (SBRT) has demonstrated promising results for patients with refractory ventricular tachycardia (VT). However, the full safety profile of this novel treatment remains unknown and very limited data from prospective clinical multicenter trials are available.

**Methods:**

The prospective multicenter multiplatform RAVENTA (radiosurgery for ventricular tachycardia) study assesses high-precision image-guided cardiac SBRT with 25 Gy delivered to the VT substrate determined by high-definition endocardial and/or epicardial electrophysiological mapping in patients with refractory VT ineligible for catheter ablation and an implanted cardioverter defibrillator (ICD). Primary endpoint is the feasibility of full-dose application and procedural safety (defined as an incidence of serious [grade ≥ 3] treatment-related complications ≤ 5% within 30 days after therapy). Secondary endpoints comprise VT burden, ICD interventions, treatment-related toxicity, and quality of life. We present the results of a protocol-defined interim analysis.

**Results:**

Between 10/2019 and 12/2021, a total of five patients were included at three university medical centers. In all cases, the treatment was carried out without complications. There were no serious potentially treatment-related adverse events and no deterioration of left ventricular ejection fraction upon echocardiography. Three patients had a decrease in VT episodes during follow-up. One patient underwent subsequent catheter ablation for a new VT with different morphology. One patient with local VT recurrence died 6 weeks after treatment in cardiogenic shock.

**Conclusion:**

The interim analysis of the RAVENTA trial demonstrates early initial feasibility of this new treatment without serious complications within 30 days after treatment in five patients. Recruitment will continue as planned and the study has been expanded to further university medical centers.

**Trial registration number:**

NCT03867747 (clinicaltrials.gov). Registered March 8, 2019. Study start: October 1, 2019.

**Supplementary Information:**

The online version of this article (10.1007/s00066-023-02091-9) contains supplementary material, which is available to authorized users.

## Introduction

In patients with structural heart disease, ventricular tachycardia (VT) plays a decisive role in sudden cardiac death [1, 2]. The treatment of patients with VT consists of antiarrhythmic medication and implantation of an implantable cardioverter defibrillator (ICD) which detects and terminates VT by antitachycardia pacing or shock. For patients with symptomatic sustained monomorphic VT despite antiarrhythmic drug therapy, catheter ablation is standard of care [1, 2]. However, catheter ablation of VT has its limitations due to comorbidities, prior procedures, or location of the VT substrate leading to approximately 20–40% recurrences even after repeat ablation [1–3]. For these patients, who often suffer from continued ICD shocks, stereotactic body radiotherapy (SBRT) [4] delivered in a single session to the arrhythmogenic substrate, also called stereotactic arrhythmia radioablation/modulation (STAR) or cardiac radiosurgery, may offer a treatment alternative for this otherwise untreatable and refractory condition [5].

Cardiac SBRT is a complex interdisciplinary treatment which requires great clinical experience in spatial VT substrate localization from electroanatomic mapping (EAM) and stereotactic radiotherapy of multidimensionally moving targets within and surrounded by highly critical organs. While cardiac SBRT can technically be performed with current modern stereotactic radiotherapy systems and adequate motion-management strategies [6], the exact radiobiological mechanisms in preexisting fibrosis and arrhythmogenic microenvironments are still not fully understood. Historical and recent preclinical and clinical data suggest that cardiac SBRT at lower doses (20–25 Gy) may induce cardiac conduction reprogramming with increased myocardial contraction while higher doses (> 30 Gy) may induce transmural fibrosis [7–10].

The first cardiac SBRT treatment for VT was performed in 2012 in Stanford (USA) [11], while the first case series in the USA and the first treatment in Germany were reported in 2017 and 2019, respectively [12, 13]. Recently, many additional case reports and small case series have been published and summarized in systematic and comprehensive reviews [14, 15]. The first prospective report in 2019 from the ENCORE-VT trial (St. Louis, USA) with 19 patients revealed a VT burden reduction of more than 75% in 89% of the patients after cardiac SBRT [16]. Furthermore, the rate of patients requiring dual antiarrhythmic medication could be reduced from 59% to 12%, with improvements in quality of life. On the other hand, severe short-term (within 90 days) adverse events (grade 3) were observed in 10.5% of the patients [17]. Recent initial reports from a single-center clinical trial (Milan, Italy) and from harmonized multicenter compassionate-use treatments (UK) showed similar results [18, 19]. While the encouraging evidence for the efficacy and safety of cardiac SBRT for VT is increasing, there are currently no prospective data demonstrating multicenter transferability of this novel treatment into a clinical trial environment.

## Methods

### Study design

The RAVENTA study is a single-arm multicenter multiplatform clinical feasibility trial that investigates cardiac SBRT for patients with refractory VT with limited treatment options. The detailed rationale and study protocol have been published previously [20]. The RAVENTA study has received approval from the German National Radiation Protection Authority (*Bundesamt für Strahlenschutz*, BfS; reference number Z5-22463/2-2018-054), from the leading ethics committee at the Christian-Albrechts-University Kiel (reference number 555-18), and from the respective local ethics committees for each participating center separately. The clinical trial is monitored by the Center for Clinical Trials in Lübeck, Germany (ZKS Lübeck, Germany; protocol number ZKS-121-09). The first five patients in the RAVENTA study were treated sequentially in each center. Each case was reviewed by an independent data and safety monitoring board (DSMB) based on detailed case reports and primary endpoint assessment [20].

### Patient selection and study endpoints

In the RAVENTA study, patients with refractory VT who are ineligible for catheter ablation at the treating university medical center are treated with cardiac SBRT focused to the underlying VT substrate. The inclusion criteria for the RAVENTA study are

- patients with structural heart disease and ICD
- age ≥ 18 years
- life expectancy > 6 months
- symptomatic monomorphic VT refractory to antiarrhythmic medication therapy at maximum tolerated dose and
  - recurring with at least three episodes within 3 months prior to inclusion or
  - inducible by ICD via noninvasive programmed stimulation (NIPS) or during electrophysiology measurement, or
  - both a) and b)

The exclusion criteria for the RAVENTA study are

- no evidence of myocardial scar triggering the VT
- ICD electrode malfunction
- prior thoracic radiation therapy
- pregnancy or breastfeeding

The primary endpoint of the RAVENTA study is 30-day postprocedural safety. This endpoint was selected based on international guidelines for introducing novel treatments into clinical practice and includes feasibility (complete delivery of SBRT) and the rate of potentially treatment-related serious adverse events (grade ≥ 3). Adverse events are assessed using the Common Terminology Criteria for Adverse Events (CTCAE, version 5.0) scale and classified regarding treatment causality with a) no causal relationship, b) unlikely causal relationship, c) possible causal relationship, and d) likely or definitive causal relationship. Secondary study endpoints evaluated within the first year of follow-up are the rate of VT episodes and ICD interventions, use of antiarrhythmic medication, toxicity, quality of life, and overall survival. Due to German regulations, all patients are followed up for at least 5 years after treatment to report any treatment-related serious adverse events beyond the clinical study duration.

### Statistical hypothesis

The aim of the study is to demonstrate complete radiotherapy delivery and safety within 30 days of the procedure. We assumed that 95% of patients will fulfill this criterion. The null hypothesis of insufficient safety is reached if ≤ 70% of patients receive complete delivery or are free from possibly treatment-related serious (grade ≥ 3) adverse events. The sample size calculation resulted in a total of 20 patients. According to Simon and Fleming’s two-stage design, an interim analysis was prespecified after five patients completed the 30-day follow-up after the procedure. All cases were reviewed by the data and safety monitoring board (DSMB) after inclusion as well as after completion of the 30-day follow-up.

### Study treatment and harmonization

All radiotherapy-related RAVENTA study treatment parts followed the procedural and technical quality guidelines for cardiac and general SBRT [4–6, 21]. In brief, patients had electroanatomic mapping (EAM; not older than 30 days) as well as a contrast-enhanced multiphase electrocardiography (ECG)-gated cardiac computed tomography (CCT). The cardiac SBRT target volume (TV) was defined from combined EAM/CCT data and included the substrate causing the VT. Based on our experience with cases treated before the start of the RAVENTA trial, TV definition and transport to the radiotherapy treatment planning systems (TPS) can vary based on clinicians’ choice and experience [22]. Thus, an informal case review within the lead study group was established for the first five cases presented in this manuscript through anonymous data sharing prior to treatment. For additional quality assurance of the EAM-to-TPS transport process, dedicated data display software developed for the RAVENTA study was used (CARDIO-RT, University of Lübeck, Germany) [23].

In the TPS, the TV was enlarged to encompass cardiac motion using the CCT at end-systole and end-diastole (cardiac internal target volume [CITV]). Based on the radiotherapy system and respiratory motion compensation technique, a residual motion ITV was generated and a standardized treatment planning volume (PTV) margin (3–5 mm) was added to encompass treatment uncertainties. Treatment planning was based on the RAVENTA study benchmark [24] and the previously published study protocol [20]. Briefly, a single-fraction dose of 25 Gy was prescribed to the PTV (PTV D_95%_ ≥ 25 Gy). Organ at risk constraints have been published with the trial manuscript [20]. Study documentation was based on the International Commission on Radiation Units and Measurements (ICRU) report 91 for stereotactic treatments with small photon beams. Patient positioning and target localization for treatment was performed with on-board cone-beam CT (CBCT, *n* = 4) or stereoscopic X‑ray images (*n* = 1) using the ICD leads as landmarks in all cases.

All patients were treated with continuous ECG monitoring and a standby cardiac emergency team during treatment and were hospitalized for the procedure to ensure close monitoring after treatment. All cases were reviewed by a multidisciplinary DSMB regarding safety and the primary endpoint.

## Results

From 11/2019 to 11/2021, five patients were enrolled at the three participating centers. Baseline characteristics of these patients are summarized in Table 1. Detailed case reports of all patients are provided in the online supplemental material. Two patients each had ischemic and dilated cardiomyopathy, while the remaining patient suffered from hypertrophic cardiomyopathy. All but one patient had undergone prior catheter ablations. The patient without prior catheter ablation suffered from dilatative cardiomyopathy and catheter ablation was not deemed feasible by the treating electrophysiologist due to the location of the VT substrate deep within the thickened ventricular septum. The patients had had between 5 and 140 VT episodes within 12 weeks prior to enrolment. One patient was enrolled with inducible VT at EAM. One patient was enrolled during electrical storm while mechanically ventilated and sedated (patient 5).

**Table 1:** Patient characteristics

	1	2	3	4	5
Age (years)	74	68	67	49	63
Sex	Male	Male	Male	Male	Female
Structural heart disease	DCM	DCM	ICM	HCM	ICM
Prior catheter ablations	0	6	5	3	1
Antiarrhythmic drugs at enrolment	Amiodarone Mexiletine Bisoprolol	Amiodarone Metoprolol	Amiodarone Metoprolol	Amiodarone Mexiletine Bisoprolol	Amiodarone Mexiletine Lidocaine
VT episodes ≤ 12 weeks prior to enrolment	42 (electrical storm)	5	2 (inducible VT at EAM)	6	140 (electrical storm)
LV-EF at enrolment	35%	40%	35%	45%	20%

*DCM* dilative cardiomyopathy, *EAM* electroanatomic mapping, *HCM* hypertrophic cardiomyopathy, *ICM* ischemic cardiomyopathy, *LV-EF* left ventricular ejection fraction, *VT* ventricular tachycardia

Table 2 shows the treatment and planning parameters for the treated patients. For respiratory motion management, a residual motion ITV was generated from respiration time-resolved CT (*n* = 4) or end-inspiration CT alone (*n* = 1, gating concept with repeated deep-inspiration breath-hold [DIBH]). Median clinical target volume (CTV) was 8.1 ml (range 6.0–34.4 ml) and median PTV was 69.6 ml (range 43.4–80.7 ml). The treatment was successfully delivered in all patients and median treatment time was 20 min (range 9–61 min).

**Table 2:** Treatment characteristics

	1	2	3	4	5
Respiratory motion management	ITV	ITV	DIBH gating	ITV	ITV
Delivery technique	4 DCA	6 DCA	3 IMAT arcs	Robotic	3 IMAT arcs
Treatment time (min)	16	20	61	39	9
Image guidance	CBCT	CBCT	Breath-hold CBCT	Stereoscopic X‑ray	CBCT
TV (ml)	8.1	7.0	10.8	34.4	6.0
PTV (ml)	80.7	80.1	44.4	69.6	43.4
PTV D_98%_ (Gy)	24.0	23.4	24.6	24.7	24.6
PTV D_50%_ (Gy)	28.0	28.5	27.4	27.6	27.4
PTV D_2%_ (Gy)	29.8	29.9	29.5	29.5	29.1

*CBCT* cone-beam computed tomography, *DCA* dynamic conformal arc, *DIBH* deep-inspiration breath-hold, *IMAT* intensity-modulated arc therapy, *ITV* internal target volume, *PTV* planning target volume, *TV* target volume

At interim analysis, median follow-up after treatment was 6 months (range 1–14 months). There were seven grade ≥ 3 adverse events. However, all serious adverse events were classified as not related to the study treatment by the local investigators, which was subsequently confirmed by the data and safety monitoring board. Cardiac grade 3 adverse events included two cases of decompensated heart failure in patients with a known history of heart failure and two instances of cardiac palpitations in a patient with prior history of atrial fibrillation. One patient suffered from pneumothorax after placement of a pleural catheter for pleural effusion that was present before cardiac SBRT and had been managed with pleural drainage before (patient 5). One patient had an episode of grade 3 upper gastrointestinal bleeding and epistaxis after endoscopy (patient 1).

**Fig. 1:**
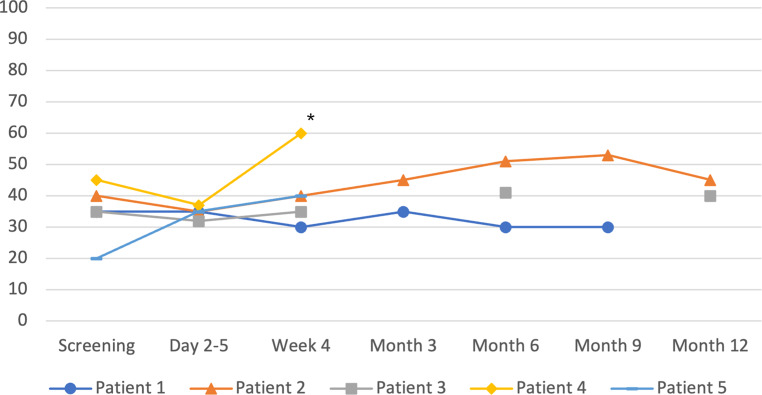
Left ventricular ejection fraction (LV-EF) during the study. *Asterisk* Patient 4 died after the 6‑week follow-up visit with recurrent ventricular tachycardia

There were only three possibly treatment-related adverse events. This included one patient with dry cough who received an ACE-inhibitor (grade 1) without signs of pneumonitis on the protocol-recommended CT scan and one patient with asymptomatic progression of preexisting mitral regurgitation (grade 2). Cardiac function as determined by left ventricular ejection fraction (LVEF) remained stable or increased during follow-up (Fig. 1). No cases of pneumonitis were detected with routine computed tomography 3 months after treatment. A complete list of adverse events is provided in the online supplemental material (Supplementary Table S1).

**Fig. 2:**
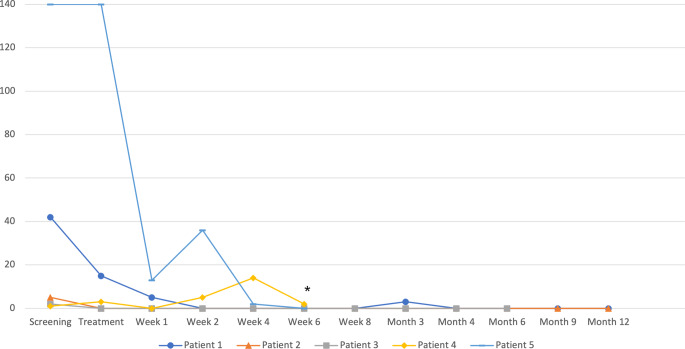
Ventricular tachycardia (VT) episodes per patient during the study. *Asterisk* Patient 4 died after the 6‑week follow-up visit with recurrent ventricular tachycardia

**Fig. 3:**
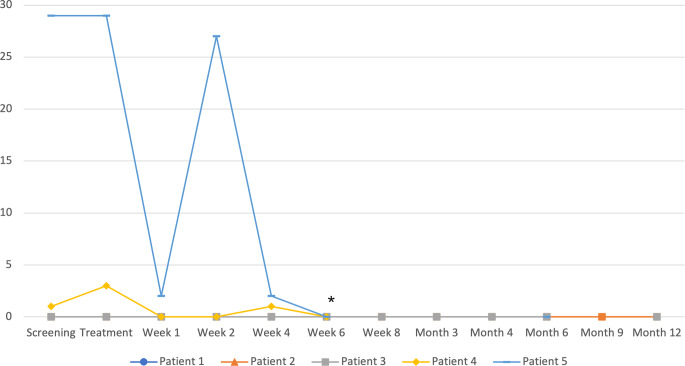
Number of implantable cardioverter defibrillator (ICD) shocks per patient during the study. *Asterisk* Patient 4 died after the 6‑week follow-up visit with recurrent ventricular tachycardia

In terms of efficacy, three out of five patients had a reduction in VT episodes (Fig. 2). ICD shocks during follow-up are shown in Fig. 3. Only two patients suffered from ICD shocks during follow-up. One patient was readmitted twice due to VT episodes 4–6 weeks after treatment (patient 4) and received adjustment of the amiodarone dose and an external cardioversion. Seven weeks after treatment, the patient was readmitted. ICD interrogation showed recurrent VT with antitachycardia pacing and ICD shocks. The general condition further deteriorated, and the patient died 5 days later in cardiogenic shock with recurrent VT and electrical storm.

One patient initially presented with electrical storm after myocardial infarction (patient 5). The patient had refractory VT despite catheter ablation, maximal antiarrhythmic drug therapy, and bilateral stellate ganglion blockade. In the first days after treatment, there was a substantial decrease in VT episodes. However, after cardiac SBRT, several new VT episodes with a different morphology compared to the original VT were noted distant to the original VT substrate. Repeat EAM 10 days after cardiac SBRT with catheter ablation in the region corresponding to the new VT morphology (segment 10) was performed, after which the VT completely receded. Due to bacteremia with vegetations on the ICD leads upon serial transesophageal echocardiography, the ICD had to be explanted 19 days after cardiac SBRT with reimplantation after 4 weeks. During the following weeks, the patient could be extubated and discharged and remained free from VT during follow-up. Patient 1 completed the trial follow-up but died 14 months after treatment from progressive heart failure. As mentioned previously, patient 4 died due to recurrent VT in electrical storm 7 weeks after cardiac SBRT. A detailed description of all cases is provided in the online supplementary material.

## Discussion

Since publication of the first case series from the group at Washington University in St. Louis (USA), there has been a growing interest in the use of cardiac SBRT for patients with refractory VT [12]. Most of the subsequent reports are retrospective case series or case reports. Three prospective single-center studies have been published so far [12, 18, 25]. There is only one multicenter case series describing the initial experience with cardiac SBRT from three centers in the UK that introduced the procedure in close collaboration with the St. Louis group [19]. For the multicenter RAVENTA study, we were able to demonstrate early initial feasibility and short-term safety for the first five patients. There were no possibly treatment-related serious adverse events in the first 30 days after treatment and preliminary data suggest a reduction in VT burden and associated ICD interventions, while efficacy and long-term safety require longer follow-up and completion of the clinical trial.

Cardiac SBRT involves interdisciplinary discussions and interactions between cardiology/electrophysiology and radiation oncology/medical physics in terms of the indication for treatment and technical feasibility, as well as target volume delineation, treatment planning, and treatment delivery. Due to the limited clinical experience with cardiac SBRT, many open questions and uncertainties remain. These are related to the efficacy as well as the safety of the procedure and we decided to designate safety as the primary endpoint of the RAVENTA study. While SBRT has been used for thoracic malignancies for over 20 years, the heart has been designated as critical organ at risk for radiotherapy in almost all cases, with the rare exception of intra- or paracardiac tumors. Thus, dose constraints for specific cardiac substructures are lacking. The current literature suggests a favorable safety profile of cardiac SBRT and a recent systematic review of 57 patients from 13 studies found 9 acute adverse events (within 90 days of the procedure) [15]. Periprocedural toxicity mostly consisted of nausea and vomiting in patients with an inferior target location. However, there were single cases of pericarditis, progressing heart failure, and stroke, as well as two cases each of pulmonary embolism and pneumonitis [12, 18, 25]. So far, the ENCORE-VT trial represents the only source of data on longer-term safety. Beyond 90 days, there were three probable treatment-related serious adverse events: two cases of pericarditis (each grade 3) and one case of gastropericardial fistula (grade 4) occurring more than 2 years after treatment. The Czech group recently reported another case of esophagopericardial fistula, ultimately resulting in death of the patient 9 months after cardiac SBRT [26]. These serious complications demonstrate the necessity for careful procedural planning and thorough follow-up within clinical trials. Due to the limited follow-up so far, the long latency for radiotherapy-related adverse events, and the retrospective design of most studies, chronic toxicity of cardiac SBRT is most likely underreported and all RAVENTA study patients will be followed in the long term for further assessment.

In terms of efficacy, the most impressive results came from the ENCORE-VT trial [16]. A reduction of ≥ 75% for VT episodes or premature ventricular contractions (PVC) was achieved in 89% of patients with a median follow-up of 13 months. Even with a median follow-up of 24 months, 78% of patients continued to meet the primary endpoint, which consisted of any reduction in VT/PVC burden [27]. Preliminary data from the single-center STRA-MI-VT trial also demonstrated a reduction in VT burden in all 7 patients after 6 months [18]. Gianni et al. on the other hand described recurrent VT in all five patients despite an initial treatment response in their prospective trial [25]. Reports from retrospective cases or case series have also suggested higher rates of VT recurrence or primary treatment failure [28–30]. In this initial report of the RAVENTA study, three out of five patients had a reduction in VT burden and ICD interventions. One patient had early appearance of a new VT morphology and received repeat EAM. Endocardial EAM showed an extensive area of low voltage (< 0.3 mV) correlating to potential scar tissue in the SBRT treatment area. One patient showed no relevant reduction of VT episodes within the initial blanking period and finally died 6 weeks after an otherwise uncomplicated STAR procedure in electrical storm.

There is only limited experience with EAM after cardiac SBRT. Gianni et al. described signs of vital myocardium upon EAM in three patients undergoing catheter ablation for recurrent VT after cardiac SBRT [25]. In one case with EAM available before and after cardiac SBRT, increased scar formation was noted in the treatment region; however, fragmented, late, and low-voltage potentials were demonstrated within this area. Kautzner et al. reported a case with early treatment failure presenting with clusters of VT recurrence and an electrical storm [31]. The EAM revealed a homogenous low unipolar voltage in the treatment area. Finally, Qian et al. published a case with recurrent VT originating from the border zone of cardiac SBRT which was intentionally spared [32]. More research is needed to determine predictors for efficacy of cardiac SBRT. Until then, expert consensus statements may aid in the optimal selection of patients [5]. Besides patient selection, determination of the target volume is a crucial step for cardiac SBRT. Two publications have elucidated pitfalls in the registration of EAM and CT images as well as in transfer of the EAM-determined target volume to the planning CT [22, 33]. Dedicated software may bridge this gap and serve as an important tool for quality assurance [23, 34, 35].

Limitations of this interim analysis report of an ongoing clinical trial include the limited follow-up as well as the number of patients treated. The clinical characteristics were quite heterogenous, ranging from one patient deemed ineligible for initial catheter ablation to patients with several prior catheter ablations as well as one patient with severe electrical storm who received treatment under sedation. Since cardiac SBRT for VT is currently regarded as a bail-out procedure, this was an expected finding. In the initial period following cardiac SBRT, assessment of efficacy is limited. Therefore, several investigators defined the first 6 weeks after treatment as the blanking period [16, 18]. Consequently, VT episodes following SBRT were defined as disease recurrence and not as adverse events. This is also in line with previous prospective trials [16, 18, 25]. Patients were recruited over a period of 2 years, which is considerably longer than initially expected and may in part be related to the COVID-19 pandemic. Data from a German hospital network suggest that hospital admissions and interventions for ventricular arrhythmias decreased by 23–27% and 21–27%, respectively, during the initial phase of the COVID-19 pandemic [36]. However, based on the interim analysis, the RAVENTA study was extended to more study centers and will continue to recruit patients with refractory VT with limited treatment options.

## Conclusion

This planned interim analysis demonstrates initial short-term safety and feasibility of cardiac SBRT in five patients with refractory VT not eligible for catheter ablation. Enrolment in the RAVENTA study will continue as planned with opening of additional clinical trial sites.

## Supplementary Information

Supplementary Table S1: Complete list of adverse events, detailed case reports for the five RAVENTA patients
