# Supplementary material for: Radiosurgery for ventricular tachycardia (RAVENTA): interim analysis of a multicenter multiplatform feasibility trial
Source: Strahlenther Onkol. 2023 Jun 7;199(7):621–30. doi: 10.1007/s00066-023-02091-9 (PMC10245341; doi:10.1007/s00066-023-02091-9)
Supplement: Supplementary file 1 — Supplementary Table S1: Complete list of adverse events, detailed case reports for the five RAVENTA patients [file 66_2023_2091_MOESM1_ESM.docx]

**Supplementary Material**

**Supplementary Table S1: Complete list of adverse events**

| **Patient** | **Time between treatment and onset (days)** | **Adverse event** | **CTCAE-grade** | **Relation-ship with study treatment** | **Treatment of AE** |
| --- | --- | --- | --- | --- | --- |
| 1 | 42 | Orthostatic dysregulation | 1 | not likely | None |
| 1 | 63 | cardiac decompensation | 3 | none | Diuretics |
| 1 | 122 | cardiac decompensation, acute on chronic kidney failure | 3 | none | Hydration |
| 1 | 333 | Upper gastrointestinal bleeding | 3 | none | Preexisting angiodysplasia, endoscopy with clipping |
| 2 | 8 | Atrial fibrillation | 3 | not likely | Increase of Amiodarone-dose |
| 2 | 103 | Atrial fibrillation | 3 | not likely | Increase of Amiodarone-dose |
| 4 | 10 | Dry cough | 1 | possible | Change of Ramipril to Candesartan |
| 4 | 26 | Recurrent VT leading to hospitalization | 3 | none | External cardioversion |
| 4 | 32 | Mitral regurgitation | 2 | possible | None |
| 4 | 46 | Recurrent VT (electrical storm) | 5 | none |  |
| 5 | 2 | Pneumothorax | 3 | none | Thoracic drainage |
| 5 | 14 | Sepsis | 4 | none | Antibiotics |
| 5 | 38 | Swallowing dysfunction | 2 | none | Placement of percutaneous endoscopic gastroenterostomy |
| 5 | 45 | Pneumonia | 2 | none | Antibiotics |

**Supplementary detailed case descriptions**

**Case 1**

A 74-year-old male patient with dilatative cardiomyopathy and a history of coronary artery disease presented with recurrent slow ventricular tachycardia (VT). He had undergone placement of an implantable cardioverter defibrillator (ICD) 7 years earlier, which was replaced some months before presentation. He had a history of mild mitral and severe tricuspid regurgitation.


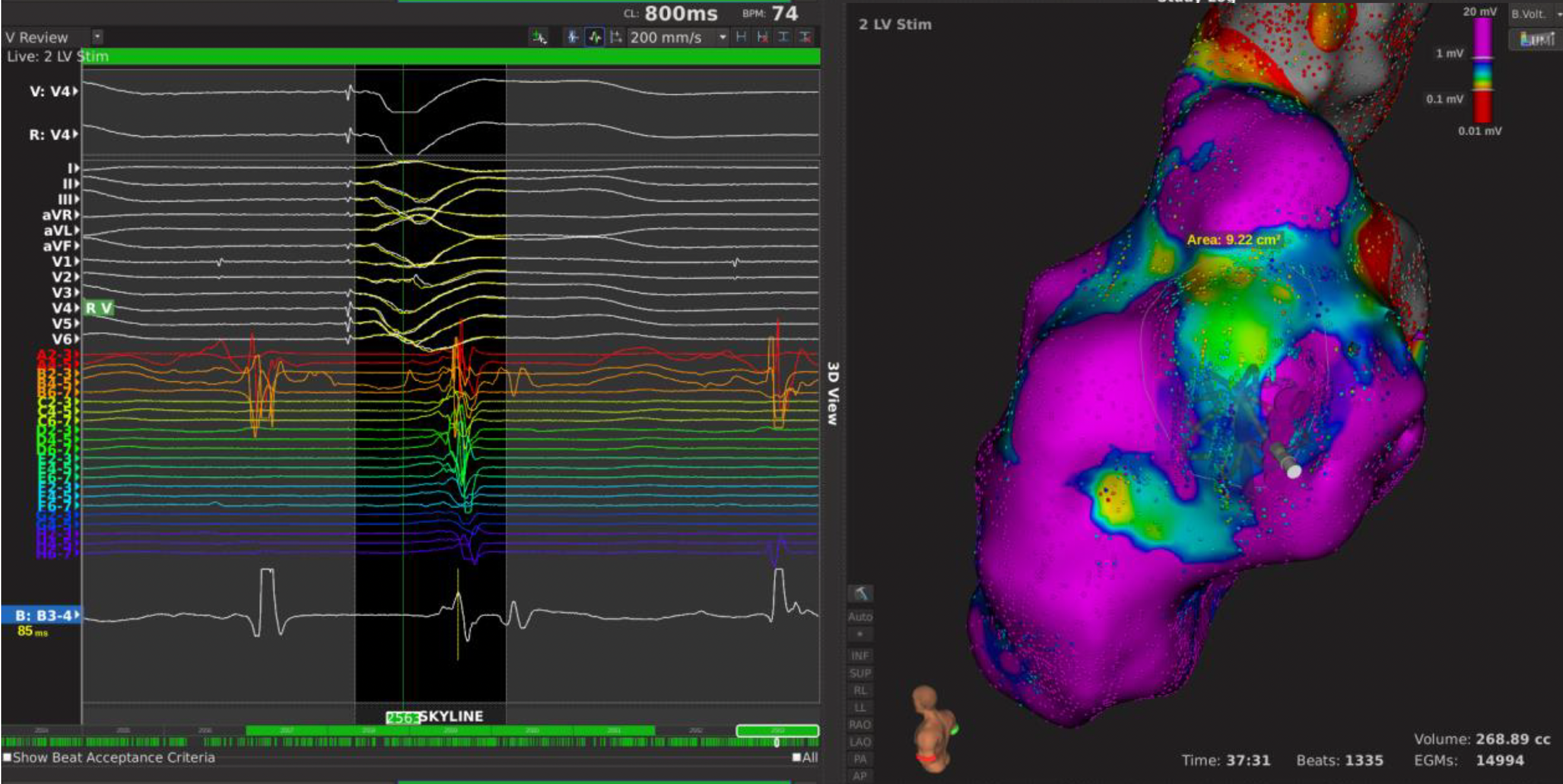


Figure 1: Electroanatomical mapping for patient 1.


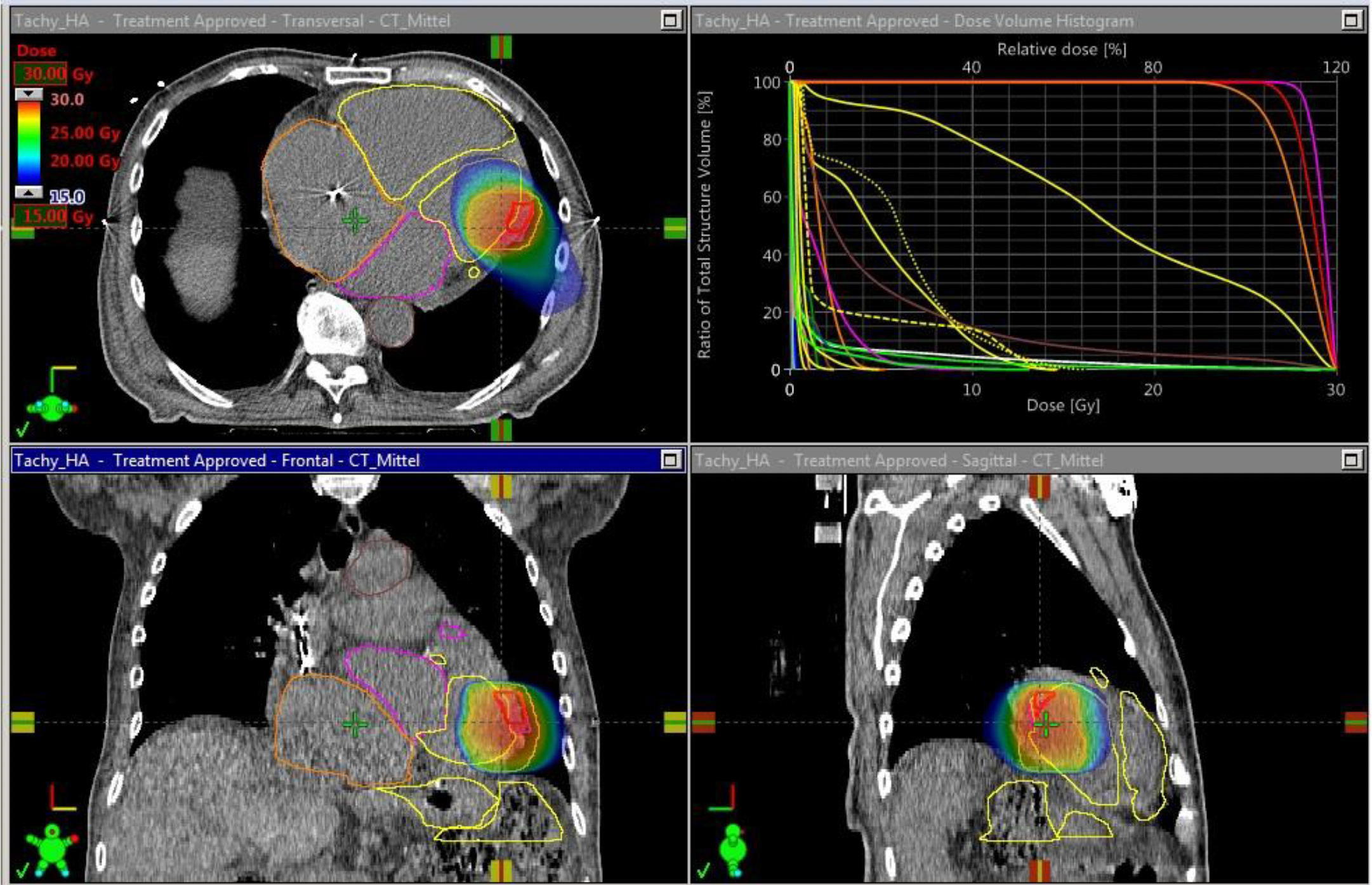


Figure 2: Treatment plan for patient 1.

At presentation, he received amiodarone (200 mg b.i.d.), bisoprolol (5 mg b.i.d + 2.5 mg) and mexiletine (300 mg three times per day). Electroanatomical mapping revealed the lateral left ventricular wall as the source of the clinical VT. Catheter ablation was not deemed feasible by the treating electrophysiologist.

The patient underwent treatment with 4 dynamic conformal arcs (True Beam STX, Varian Medical Systems) without any periprocedural adverse events.

The course after cSBRT was uneventful, the patient was discharged 7 days after the procedure. During follow up, the patient had 3 VTs recorded 3 months after treatment, all terminated by anti-tachycardia pacing (ATP). At final follow up, the patient was free from VT. Left ventricular ejection fraction was stable between 30 and 35%. In terms of adverse events (AE), the patient had two episodes of grade 3 upper gastrointestinal bleeding due to gastric angiodysplasia as well as transient orthostatic dysregulation (grade 1), all of which were rated as non-treatment related AEs.

**Case 2**

A 68-year-old male presented with recurrent VT. He was diagnosed with dilative cardiomyopathy three years earlier and received placement of an ICD after cardiac arrest. The ICD was changed for an ICD with cardiac resynchronization 3 months prior to treatment. He had undergone 6 prior catheter ablations. He had a history of atrial flutter and atrial fibrillation with prior atrial isthmus ablation and pulmonary vein isolation. The patient received treatment with amiodarone 200 mg o.d. and metoprolol 95 mg b.i.d.

Electro-anatomical mapping revealed a region in the interventricular septum as the likely cause of the clinical VT. The patient underwent treatment with 4 dynamic conformal arcs (True Beam STX, Varian Medical Systems) without any periprocedural adverse events.


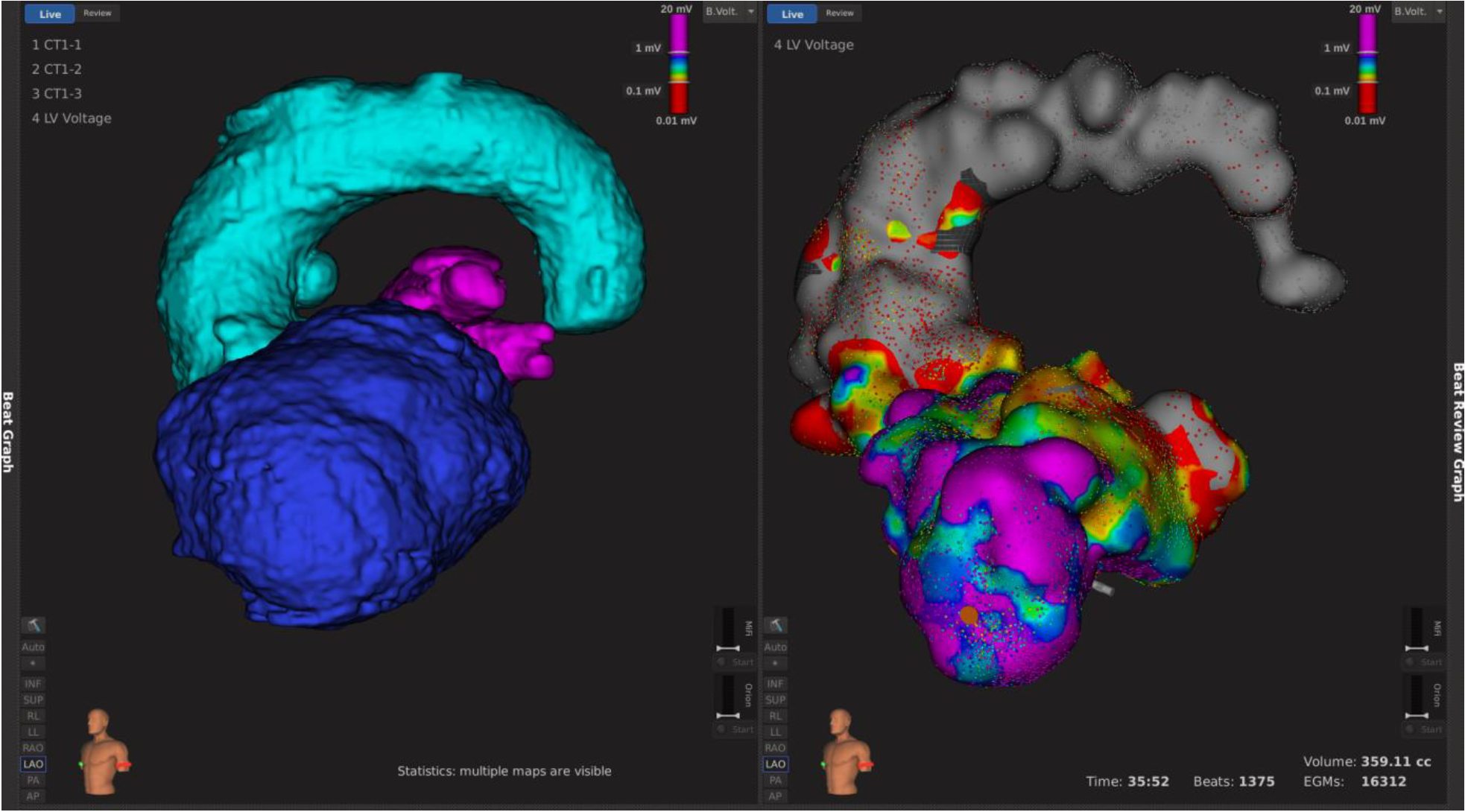


Figure 3: Electroanatomical mapping for patient 2 in LAO-view.


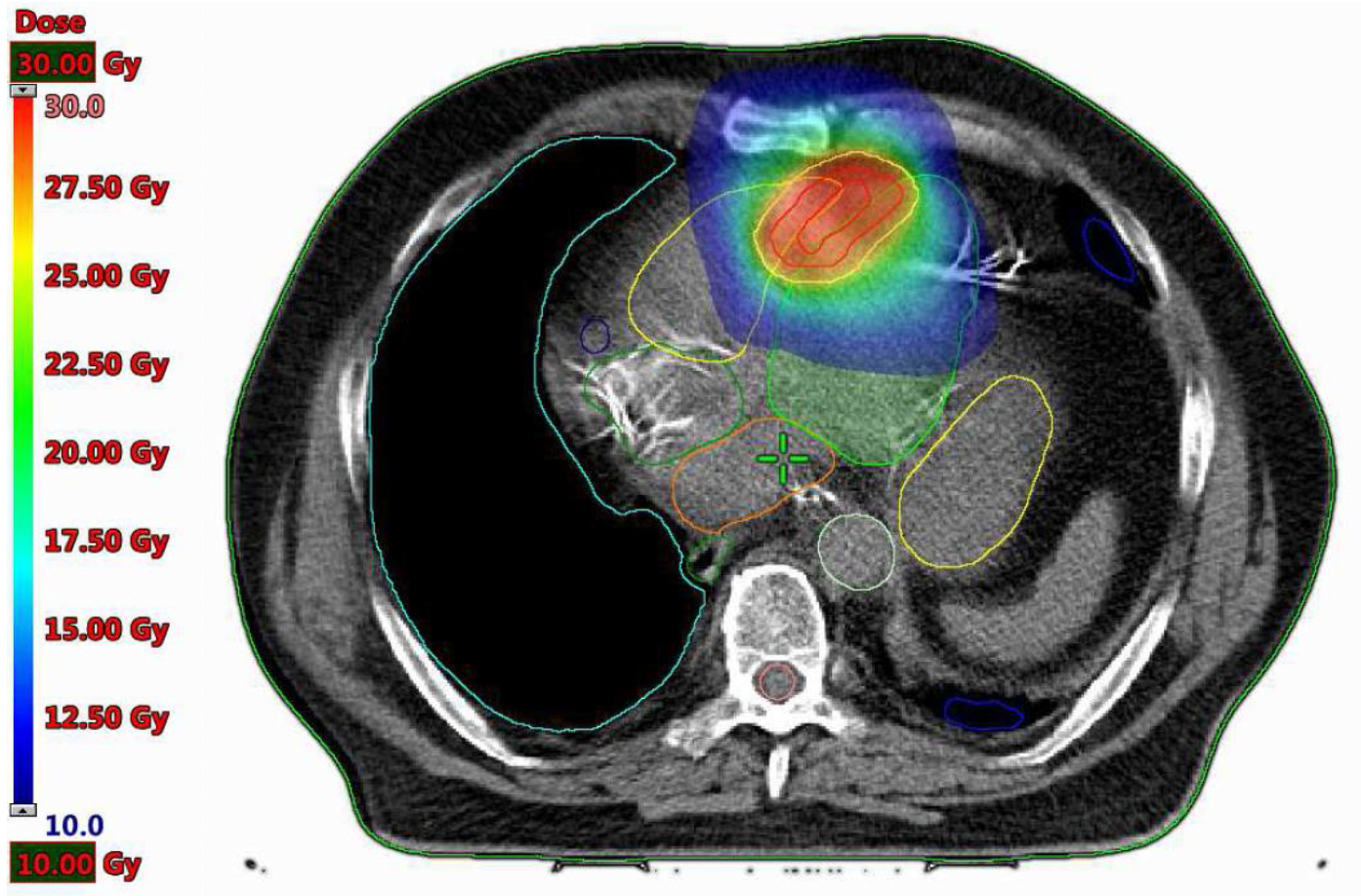


Figure 4: Treatment plan for patient 2.

At the regular study visit 4 days after the procedure, episodes of atrial fibrillation with associated supraventricular tachycardia were detected. The patients was discharged on the same day. Eight days after the procedure, the patient was re-admitted for palpitations related to atrial fibrillation with supraventricular tachycardia. The dose of amiodarone was increased to 200 mg b.i.d after which the tachycardia subsided. The patient was discharged three days later. Due to the prior history of atrial fibrillation, this event was rated as non-treatment related.

Until final follow up, no VTs were detected. LV-EF slightly increase from 40-45% to 50%.

**Case 3**

A 67-year-old male presented to the outpatient clinic due to symptomatic palpitations. ICD interrogation showed a documentation of sustained VT (cycle length 370-400 ms) effectively treated with anti-tachycardia pacing.

The patient has a history of ischemic cardiomyopathy and received an ICD for secondary prevention in 2008. He was ablated for recurrent VT five times between 2014 and 2020. Coronary angiography did not reveal any relevant coronary artery stenosis. The left-ventricular ejection fraction was 35-40%. Non-invasive ventricular stimulation induced the clinical tachycardia (TCL 330 ms, S1: 320 ms) reproducibly. 12-lead ECG was then documented, and VT treated with ATP. Inferobasal region (segment 4) was identified and chosen for SBRT in DIBH. The patient received a single fraction with 25 Gy and could be discharged without any acute side effects of the treatment.


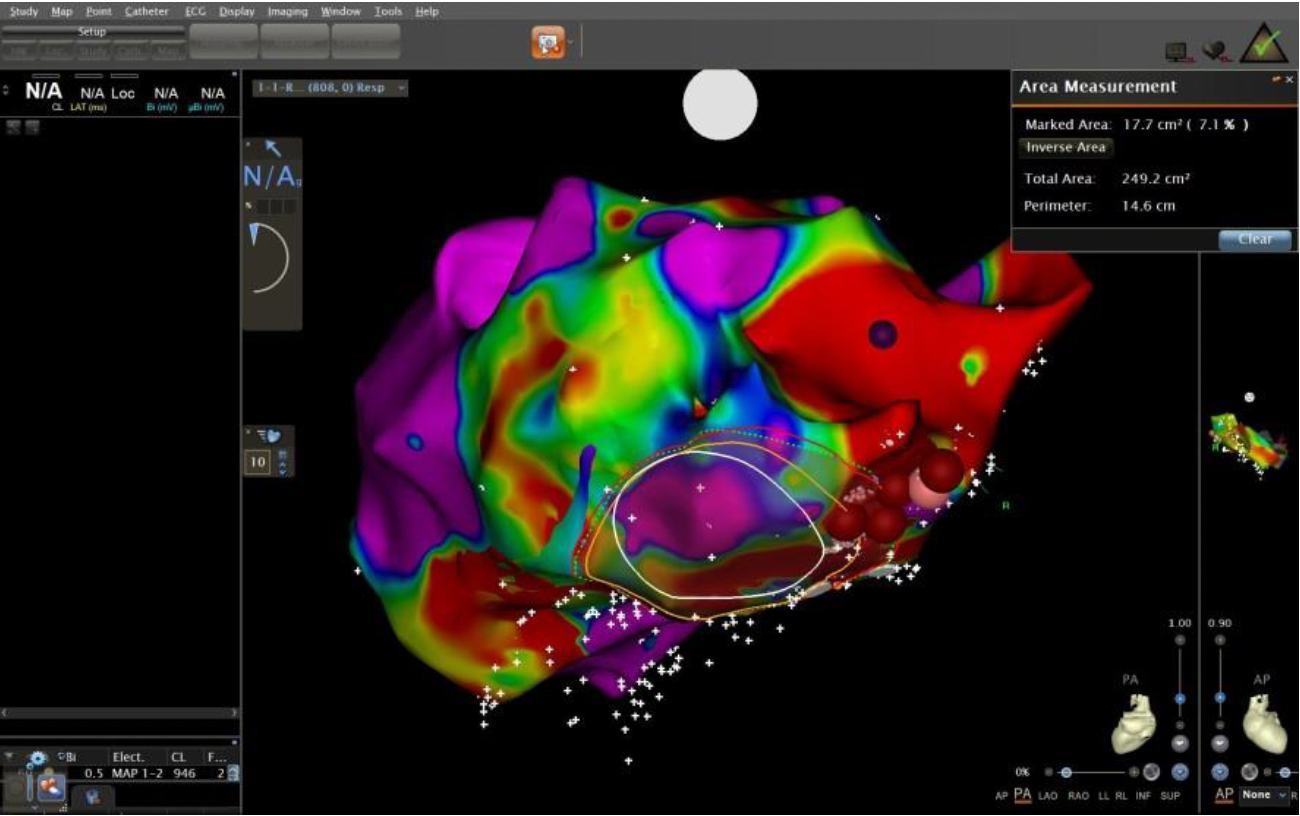


Figure 5: Electroanatomical mapping for patient 3 in PA-view.


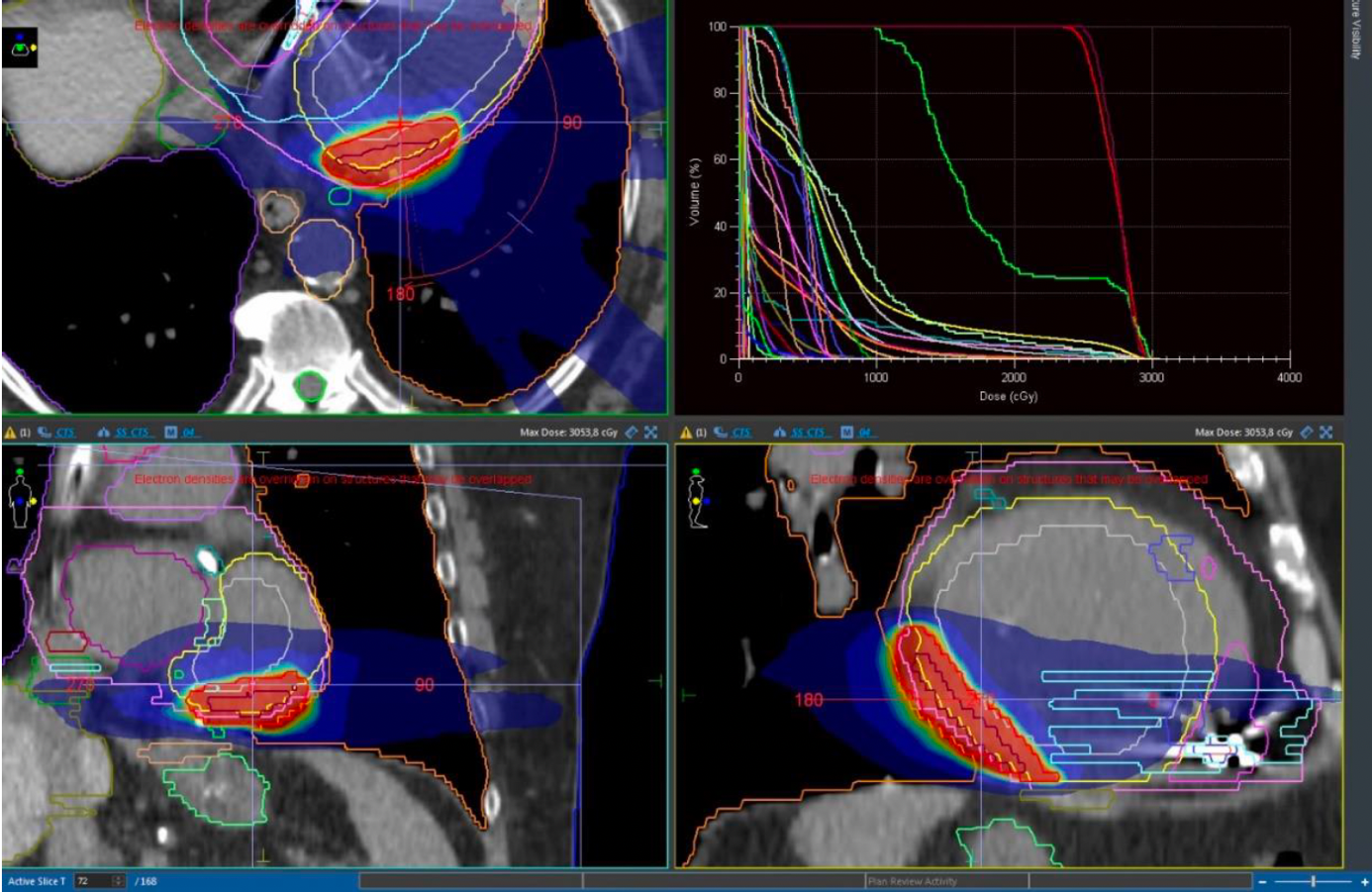


Figure 6: Treatment plan for patient 3.

The follow-up in the framework of the study was uneventful. Within one year we did not observe any VT recurrence (neither sustained, nor non-sustained) in the ICD interrogation.

**Case 4**

A 49-year-old male patient with a longstanding history of hypertrophic cardiomyopathy (first diagnosed in 2014) presented with recurrent VT. In 2018, the patient had received an implantable cardioverter defibrillator (ICD) and was treated with amiodarone following a sustained ventricular tachycardia with consecutive cardiogenic shock. In 2019, catheter ablation was performed after occurrence of another episode of VT for several VT-morphologies. Anti-arrhythmic drug therapy (AADT) was escalated to amiodarone 200mg bid and metoprolol 95mg bid. A cardiac MRI showed extensive intraseptal fibrotic areas and in the electrophysiological examination VT was still inducible. AADT was extended to mexiletine 3x 200mg/day. The patient reacted with slow VTs below the detection zone of the ICD and repeated cardiac decompensation. Heart transplant was discussed, however the patient refused to be listed. In March 2020, he was hospitalized once more during an episode of electric storm and presented multiple ICD shocks following slow VTs with acceleration to tachycardia.


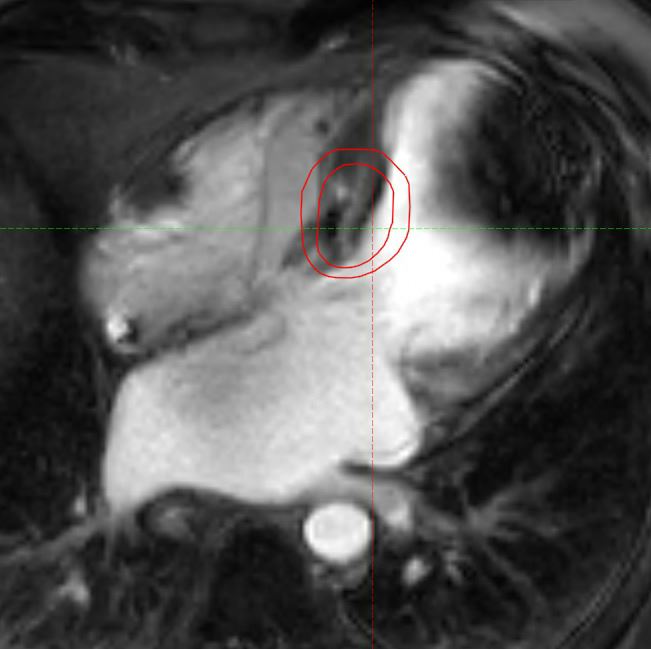

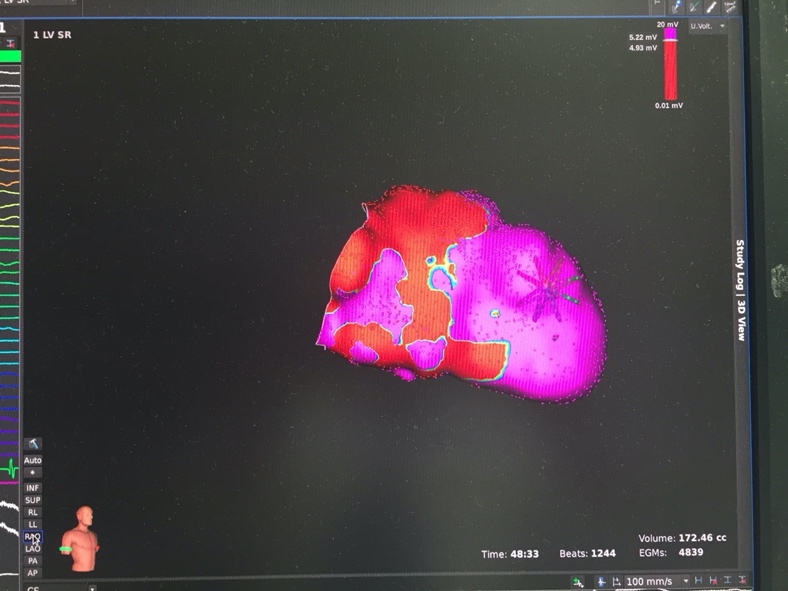


Fig. 7a MRI with late enhancement. Fig. 7b Electro-anatomical mapping in RAO-view.

In May 2020 the patient once again underwent electro-anatomical mapping, a reentry circuit in the basal interventricular septum was identified as the source of recurrent VT. This region, which is corresponding with the above-mentioned fibrotic area in the MRI, was chosen as target for an attempt of cardiac SBRT. Treatment was performed with a Cyberknife VSI ^TM^ using an ITV-concept based on a 4D-planning CT for motion management as tracking of the ICD-probe did not give stable results. Radioablation treatment was performed in a single session with 25 Gy applied to the 95% isodose and a dose maximum of 30.08Gy. Treatment course and short-term follow-up were uncomplicated, the patient did not present any acute treatment toxicities.

The patient was discharged home three days following treatment in unremarkable condition, no ICD-dysfunction, no ECG-abnormalities or echocardiographic changes.

During the first 2 weeks post-treatment the patient presented with several episodes of ventricular tachycardia upon ICD-interrogation (4 VTs with adequate treatment by anti-tachycardic pacing (ATP) of the ICD. 20 days and again 26 days following SBRT the patient was hospitalized with symptoms of acute heart failure, the ICD showed repeated and sustained VTs which could not sufficiently be terminated by ATP; escalation of amiodarone therapy and external cardioversion could finally stabilize the patient. Echocardiography showed a moderate increase in mitral regurgitation which was judged as an adverse event (CTCAE grade 2) with possible relationship to treatment.


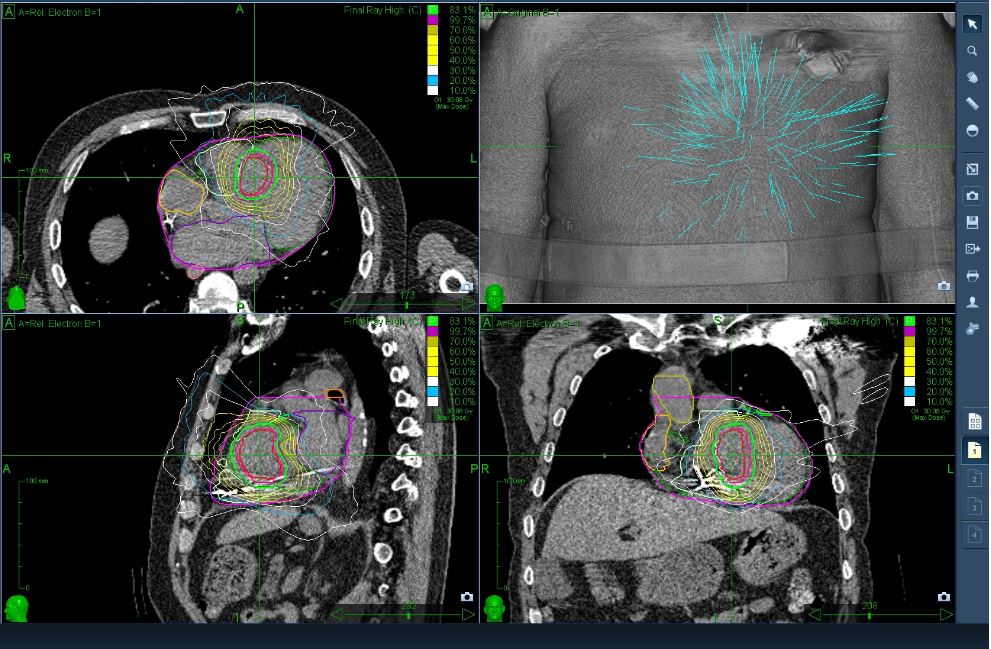


Fig. 8: Treatment plan for patient 4.

6 weeks following treatment the patient was admitted to the hospital with clinical symptoms of a stroke; ICD-interrogation revealed repeated (four) episodes of VT treated by a series of ATPs during the last three days before hospitalization. During the cause of hospitalization, the patient experienced another episode of electrical storm with a series of nine ineffective ICD-shocks and finally termination of the running VT with the tenth shock. Finally, 7 weeks and three days following SBRT the patient died after yet another episode of electrical storm with a total of 19 ineffective ICD-shocks and most likely terminally resulting pulseless electric activity.

**Case 5**

63-year-old female was admitted due to subacute myocardial infarction. After coronary angioplasty and stent implantation in the right coronary artery she developed a cardiogenic shock and was intubated. Inotropes were started (Dobutamine, Levosimendan) for treatment of the acutely decompensated congestive heart failure and she developed episodes of sustained ventricular tachycardia treated with external cardioversion. After stabilization, amiodarone and ajmaline were introduced and the patient received a DDD-AICD and further revascularization of circumflex artery and left anterior descending stenosis. Due to recurrent VTs, a radiofrequency ablation was performed targeting inferobasal and midventricular septal late potentials. Despite initial non-inducibility of clinical VTs at the end of the ablation procedure, the patient developed recurrent sustained VTs over the course of time. Mexiletine and lidocaine were then introduced, which suppressed the rate of VT occurrence without total abolishment. 12-lead ECGs of the dominant VT pattern were analyzed and segment 2 and segment 1 were identified as exit-sites of the clinical VT. The patient received a single dose radiation with 25 Gy. After SBRT VT burden gradually decreased, however a second VT morphology repeatedly occurred, now originating from the mid inferior septum and the septal apex (segments 9 and 14). Another RF ablation was undertaken which successfully treated the second VT pattern. Interestingly, 10 days after SBRT, endocardial electroanatomic mapping showed extensive area of low voltage (<0.3mV) correlating to an endocardial scar in segments 2 and 1 treated with SBRT. The antiarrhythmic drug therapy was deescalated from amiodarone, lidocaine, beta blocker and mexiletine to amiodarone and beta blocker. During her in-hospital treatment the patient developed fever and bacteriaemia and defibrillator lead-associated endocarditis was assumed. The ICD system was successfully extracted, and the patient received antibiotic treatment prior to an ICD reinsertion four weeks later. The patient remained free of VT during the further follow-up.


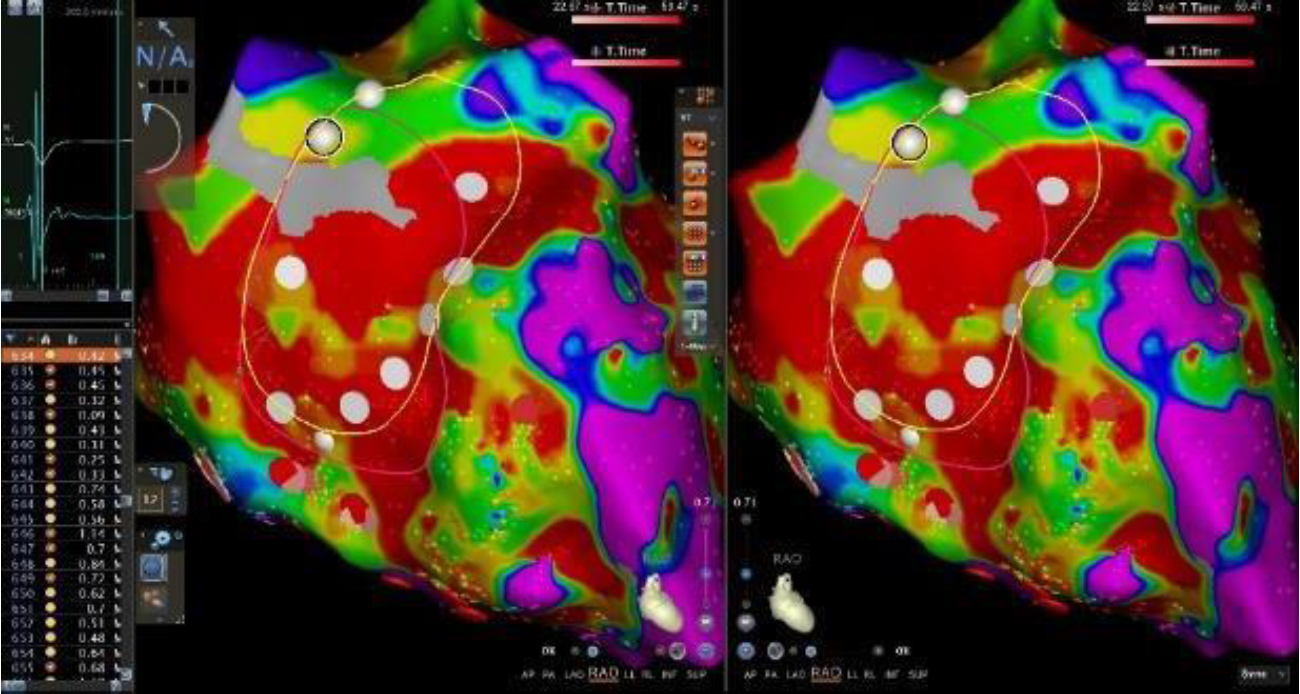


Figure 9: Electroanatomical mapping for patient 5.


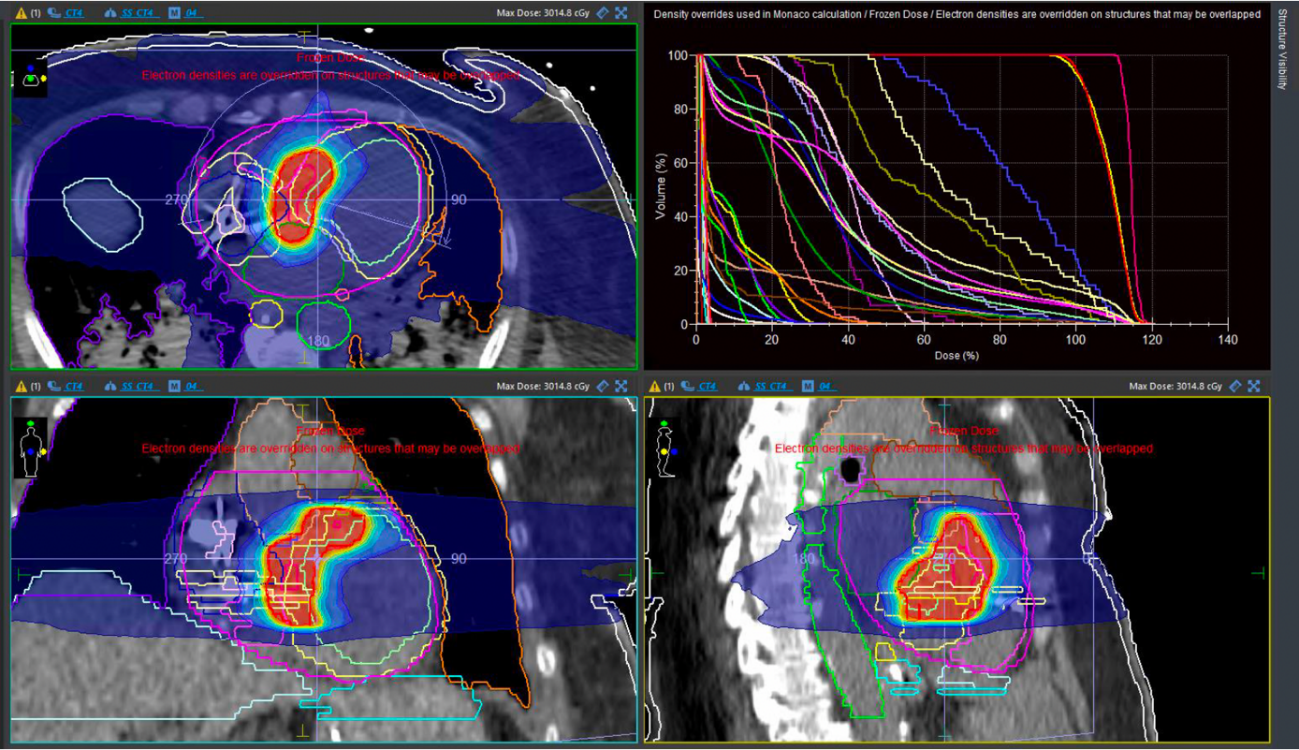


Figure 10: Treatment plan for patient 5.
